# Acetaminophen Modulates the Transcriptional Response to Recombinant Interferon-β

**DOI:** 10.1371/journal.pone.0011031

**Published:** 2010-06-09

**Authors:** Aaron Farnsworth, Anathea S. Flaman, Shiv S. Prasad, Caroline Gravel, Andrew Williams, Carole L. Yauk, Xuguang Li

**Affiliations:** 1 Centre for Vaccine Evaluation, Biologics and Genetic Therapies Directorate, Health Canada, Ottawa, Ontario, Canada; 2 Environmental Health Science and Research Bureau, Health Canada, Ottawa, Ontario, Canada; University of Maryland School of Pharmacy, United States of America

## Abstract

**Background:**

Recombinant interferon treatment can result in several common side effects including fever and injection-site pain. Patients are often advised to use acetaminophen or other over-the-counter pain medications as needed. Little is known regarding the transcriptional changes induced by such co-administration.

**Methodology/Principal Findings:**

We tested whether the administration of acetaminophen causes a change in the response normally induced by interferon-β treatment. CD-1 mice were administered acetaminophen (APAP), interferon-β (IFN-β) or a combination of IFN-β+APAP and liver and serum samples were collected for analysis. Differential gene expression was determined using an Agilent 22 k whole mouse genome microarray. Data were analyzed by several methods including Gene Ontology term clustering and Gene Set Enrichment Analysis. We observed a significant change in the transcription profile of hepatic cells when APAP was co-administered with IFN-β. These transcriptional changes included a marked up-regulation of genes involved in signal transduction and cell differentiation and down-regulation of genes involved in cellular metabolism, trafficking and the IκBK/NF-κB cascade. Additionally, we observed a large decrease in the expression of several IFN-induced genes including Ifit-3, Isg-15, Oasl1, Zbp1 and predicted gene EG634650 at both early and late time points.

**Conclusions/Significance:**

A significant change in the transcriptional response was observed following co-administration of IFN-β+APAP relative to IFN-β treatment alone. These results suggest that administration of acetaminophen has the potential to modify the efficacy of IFN-β treatment.

## Introduction

Type I interferons (IFNs) are key cytokines in the activation of the innate immune response and induce a cascade of anti-viral, anti-proliferative and immunomodulatory responses (as reviewed in [1], [2]). Briefly, Type I interferons signal through the coordinated activation of Janus (JAK) family kinases and Signal Transducers and Activator of Transcription (STAT) family members. IFN-mediated signal transduction begins when the IFN Alpha receptor (IFNAR) is dimerized by IFN, resulting in auto-phosphorylation of receptor-associated JAK tyrosine kinases. Phosphorylated JAKs activate associated STAT proteins which translocate to the nucleus where they induce transcription through binding to different promoter elements. Additionally, IFN-receptor dimerization can directly activate PI 3-kinase/Akt, Raf-1/ERK and p38 MAP kinase pathways. Beyond the induction of transcription through IFN activity the convergence of signals with other pathways such as the IκK/NF-κB can modify the transcriptional response. For example, IκK-related kinases are required for activation of Interferon Regulatory Factors (IRFs) 3 and 7 while some promoters require both NF-κB and IRF-3 binding [3], [4].

In some instances native IFN signalling is insufficient to induce the necessary immune response. It has been repeatedly demonstrated that exogenously supplied interferon can further stimulate transcription, overcoming a previously moribund response. Due to the pleiotropic effects induced by IFNs, recombinant interferon therapy has been approved to treat a variety of medical conditions including viral infections, various cancers and autoimmune disorders, including multiple sclerosis (MS) [5], [6], [7]. Treatment of relapsing-remitting MS cases with recombinant IFN-β can induce a broad shift in the auto-immune response, changing it from primarily a cytotoxic-Th1 to a humoral-Th2 response, and shifting its focus from the central nervous system to the periphery [8]. While a Th1-driven response can be an essential component of the immune response to intracellular pathogens, it leads to a significant increase in cell lysis and is of primary concern in auto-immune diseases.

Given the potent immunoregulatory role of interferon-subtypes, it is not surprising that recombinant interferon therapy has a number of common side effects including flu-like symptoms and injection-site reactions that can result in dose limitations or cessation of treatment [9]. In order to alleviate undesired side effects of IFN therapy, patients are routinely advised to self-medicate with analgesics such as acetaminophen (APAP) [10].

Acetaminophen (APAP) is an antipyretic and analgesic which is commonly used for the relief of fever, headaches and flu-like symptoms. The effects of APAP are mediated through inhibition of prostaglandin H_2_ synthase (PGHS), but this activity does not affect all cells and tissues equally [11]. While endothelial cells and neurons are extensively affected by APAP, little change is observed in other cell types such as platelets or activated leukocytes [11].

The casual use of APAP has raised concerns due to its potential to effect changes in the desired immune response, although it is not an anti-inflammatory medication. APAP administration reduces antibody production and B-cell activation in mice indicating that APAP modifies specific arms of the immune system [12]. APAP has also been observed to diminish vaccine efficacy during childhood immunizations. Children who were administered prophylactic APAP during immunization had lower mean antibody titers and less frequent vaccine responses [13]. Interestingly a similar study examining an elderly cohort found no difference in the response to an influenza vaccine when co-administered with APAP [14]. Prolonged illness, virus shedding and decreased antibody responses are associated with continual APAP treatment during rhinovirus infections indicating an effect of APAP on the host antiviral response [15]. The apparent systematic depression of aspects of the humoral immune response following APAP treatment raises questions as to whether APAP can interfere with the response to recombinant interferon therapy. Ozaki *et al* demonstrated that the induction of indoleamine 2,3-dioxygenase by IFN-α was substantially reduced when administered with APAP [16]. *In vitro*, APAP has been shown to diminish the IFN-induced antiviral response of cultured mammalian cells [17]. However, investigations into the combination IFN-α and APAP in human volunteers found no evidence for a change in either the antiviral activity or in the production of 2′5′ oligoadenylate synthetase (OAS2) from isolated PBMCs [18], [19]. The discrepancies between these observed effects may be due to APAP having a limited effect on some cell populations or conversely affecting only specific genes or proteins.

DNA microarrays allow the transcription profile of the entire genome to be assessed. They have been extensively employed to examine the transcriptional changes induced by recombinant IFN or APAP on cell lines, purified cell types, and whole tissues. Given seeming discrepancies between various studies on possible interference between APAP and the IFN-response we felt that an exploratory investigation into whether APAP is capable of modifying just the transcriptional response induced by IFN could be fruitful. Since changes induced by APAP may occur only on a few genes out of the wide number altered by IFN-β we felt that this technology would be an excellent tool to study the effects of co-treatment. The primary aim of this study was to determine how a single, sub-toxic dose of APAP alters the profile of IFN-β induced gene transcription.

## Materials and Methods

### Animals

Adult male CD-1 mice (Charles River Laboratories, Montreal, QC) from 6–8 weeks old were used in all experiments. Animals were housed in autoclaved cages in a pathogen-free environment for 1 week prior to use. Mice were provided access to autoclaved food (Purina Lab Chow #5001) and water. All experiments were conducted according to CCAC guidelines and approved by Health Canada's Animal Care Committee.

### Animal Protocol

Prior to treatment mice were fasted overnight (12 h). Access to food was restored following I.P. administration of treatment. Mice were divided into four groups of 12 and given one of following treatments: 1) vehicle control (25 mM Hepes, pH 6.0, 500 mM NaCl, 6% glycerol, 0.5% ethanol, 0.05% mouse serum albumin; 2) APAP (Sigma-Aldrich Canada, Oakville, ON) (300 mg/kg) dissolved in vehicle; 3) IFN-β (3.75×10^7^ U/kg) dissolved in vehicle; 4) APAP (Sigma-Aldrich Canada, Oakville, ON) (300 mg/kg)+IFN-β (PBL Biomedical Laboratories, Piscataway, NJ) (3.75×10^7^ U/kg) dissolved in vehicle.

### Sera and Tissue Collection

Retro-orbital blood samples (50 µL) were collected 24 h prior to treatment. Mice were anesthetised with isofluorane 1.5 or 4 hours following treatment (6 mice per time point/treatment group). Total blood was collected by cardiac puncture. Blood samples were transferred to serum separator tubes (Becton Dickinson, Franklin Lakes, NJ) and centrifuged. Serum was frozen in liquid nitrogen and stored at −80°C until analysis. Liver lobes were cut into ≈200 mg sections, frozen in liquid nitrogen and stored at −80°C until analysis.

### Toxicity Tests

Hepatic injury was evaluated by measuring the activities of the hepatic enzymes alanine amino transferase (ALT), serum alkaline phosphatase (ALKP), aspartate aminotransferase (AST) and total bilirubin (TBil) activity in the serum using VetTest arrays on a VetTest Chemistry Analyzer (Idexx Laboratories, UK). Tests were performed according to the manufacturer's instructions.

### RNA Extraction and Purification

Total RNA was isolated from liver samples using TRIzol reagent (Invitrogen, Burlington, ON) and purified using RNeasy Spin Columns (Qiagen, Mississauga, ON). RNA quantity and an initial quality estimate were determined using a ND-1000 spectrophotometer (Nanodrop Ltd, Willmington, DE). RNA quality was then assessed using the Agilent 2100 Bioanalyzer (Agilent Technologies, Mississauga, ON) and only samples with a RNA integrity number (RIN) of 8 or greater were employed.

### Microarray Hybridization

A reference design was used for the microarray experiment [20], [21], with experimental RNA samples labelled with Cy5 and technical replicates of a commercially-available universal reference RNA sample (URR; Stratagene, La Jolla, CA) labelled with Cy3. Cyanine-labelled cRNA (Perkin Elmer Life Sciences, Waltham, Massachusetts) was produced from individual liver samples (2 µg) from 40 mice (two time points, comprised of 4 treatment groups with 5 mice each) and URR using Low RNA Input Fluorescent Amplification kits (Agilent Technologies) according to the manufacturer's instructions. Labelled RNAs were co-hybridized to Agilent 22 k oligonucleotide microarrays (Mouse V2 G4121B, Agilent Technologies) at 60°C for 17 h. Hybridized arrays were washed, fixed and scanned on a ScanArray Express (Perkin-Elmer Life Sciences) and data acquired using Imagene 5.5 (Biodiscovery Inc., El Segundo, CA).

### Statistical Analysis of Microarray Data

All pre-processing of the data was conducted using R [22], [23]. The median signal intensities were normalized using the global lowess method [23] and the transform.madata function in the MAANOVA library [24]. Ratio intensity plots were constructed for the raw and normalized data for each array and hierarchical clustering using complete and single linkages were generated to identify and exclude microarrays with poor data quality.

Differentially expressed genes were identified using the MAANOVA library by relative intensity. An ANOVA model included the main effect of treatment, time and a treatment by time interaction. The date of hybridization was used in the model as block effect. The Fs statistic [25], a shrinkage estimator, was used for the gene-specific variance components and the associated *p-*values for all the statistical tests were estimated using the permutation method (10,000 permutations with residual shuffling). These *p-values* were then adjusted for multiple comparisons by using the false discovery rate approach (FDR) [26]. The least-squares means were used to estimate the fold changes for each pair-wise comparison. The complete list of the probes used and expression analysis are submitted to Gene Expression Omnibus GEO # GSE18949. Genome-wide expression patterns of all genes with an FDR<0.05 were initially analyzed using the Pearson correlation/clustering function in GeneSpring GX10.02 (Agilent Technologies, Mississauga ON).

### DAVID Analysis

Gene Ontology analysis was performed by DAVID (Database for Annotation Visualization and Integrated Discovery, NIAID/NIH), which was used to cluster gene ontology term enrichment [27], [28]. Probes that were identified as differentially regulated during initial microarray analyses (Treatment Effect FDR≤0.05) were sorted by group (Group vs. Control FDR≤0.05) and a Fold change (+/− 1.5). These probe lists were converted to David IDs and analyzed using medium classification stringency.

### Gene Set Enrichment Analysis

Gene Set Enrichment Analysis (GSEA) of the lowess normalized data was performed using the GSEA software package [29], [30]. A ranked list of genes from the microarray data set was generated based on their metric to noise ratio. Curated biological pathway gene sets from the Molecular Signature Database (MsigDB) were screened against the ranked gene list and an order enrichment score (ES) was calculated for each gene set. This value is derived by walking along the ranked list using a cumulative sum statistic which increases when a member of a specific gene set is found in the ranked gene list and is penalized when it does not appear in the gene set [30]. The GSEA parameters used included metric, signal to noise; enrichment scoring statistic, weighted; permutation type, gene set; permutation number, 1000; and gene size restrictions, 15 minimum and 500 maximum. To adjust for multiple hypothesis testing, the ES for each gene was normalized to account for the size of the set, yielding a normalized enrichment score (NES). The proportion of false positives was controlled by calculating the false discovery rate (FDR) corresponding to each NES [26]. After running GSEA, the leading edge analysis feature of the software was used to examine the genes in the leading edge subsets of selected enriched gene sets.

### Real-Time Reverse Transcription Polymerase Chain Reaction (RT-PCR)

Taqman gene expression assays (Applied Biosystems) [Mm00516005_m1 (Hmox1), Mm01601704_g1 (Krt18), Mm00456139_m1 (Blnk), Mm00460961_m1 (Oas2) and Mm00549143_m1 (Irak2), Mm01613158_m1(EG634650), Mm01247052_m1 (Zbp1), Mm01704846_s1 (Ifit-3), Mm00455082_m1 (Oasl1), Mm01705338_s1 (ISG15)] were used to examine the relative quantity of specific RNAs in each treatment group. Total RNA (2.5 µg per sample) was reverse transcribed and quantified on an Applied Biosystems 7500 Real-Time PCR instrument (Applied Biosystems) according to the manufacturer's instructions. Beta-actin was used as an endogenous control, threshold cycles were averaged and expression levels were calculated as relative to vehicle control data.

## Results

### Experimental Design

Pilot studies were conducted in which mice were treated with varying doses of APAP or IFN-β (APAP 100–550 mg/kg, IFN-β 1×10^6^–1×10^8^ U/kg) and liver and sera were collected at several time points (1.5–24 h). During these pilot studies hepatotoxicity was evaluated by microscopic examination of formalin-fixed hepatic tissue as well as by assessment of serum ALT and AST levels. No evidence for hepatotoxicity was observed in samples collected 1.5, 4, 8 and 24 h following treatment with any drug dose (data not shown). This was expected as young CD-1 mice are highly resistant to APAP-mediated hepatotoxicity and do not suffer toxic effects when administered APAP at these doses [31], [32]. As such, we selected one dose (APAP 300 mg/kg and IFN-β 3.75×10^7^ U/kg) and two time points (1.5 and 4 h) based on the transcriptional responses of several target genes in the treatment groups as measured by RT-PCR (data not shown). These time points were consistent with previously published studies on mRNA expression following APAP or IFN-β treatment [33], [34]. RNA was extracted from the large liver lobe and applied to microarray and RT-PCR analysis as described in the Materials and Methods.

### Hepatotoxicity induced by treatment

To monitor for any overt hepatotoxic events ALT, ALKP, AST and TBil assays were performed on the pre- and post-treatment serum of CD-1 mice at 1.5 h and 4 h time points. The levels of ALT, ALKP, AST or TBil did not differ significantly in mice receiving APAP, IFN-β or IFN-β+APAP from mice in the vehicle control group or untreated mice (data not shown). As expected, no overt signs of hepatotoxicity were observed.

### Microarray Analysis

**Figure 1:**
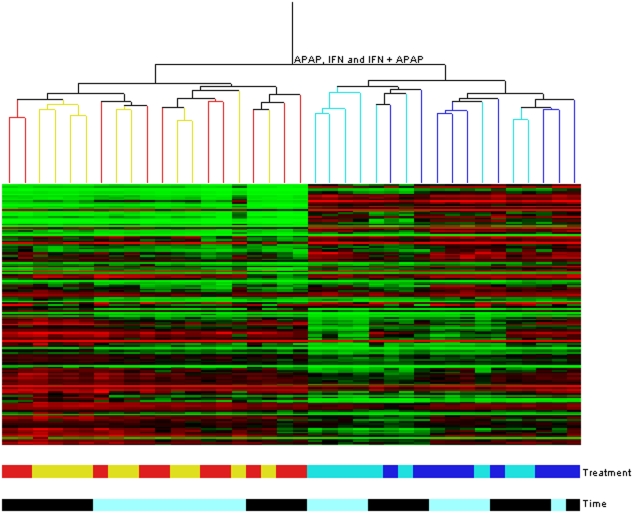
Correlation/cluster analysis of significantly altered probes. Samples clustered into two branches with vehicle and APAP treated mice on the left, and IFN-β and combined treatment on the right. Each column represents a single sample. Colours indicate high relative signal intensity (red), low relative signal intensity (green), and similar signal intensity (black) in the reference and sample channels. The branches of the tree are coloured by treatment.

Total mRNA isolated from murine hepatic tissue in mice treated with APAP alone, IFN-β alone or a combination of IFN-β+APAP were compared to those of mice treated with Vehicle alone at 1.5 and 4 hours post-treatment. Five samples were analyzed per treatment group (GEO # GSE18949). We identified 1900 probes that detected a significant change in transcript levels (FDR-adjusted *p-*value of less than 0.05) due to one or more treatments relative to the vehicle control. A correlation/cluster analysis was applied to all significant genes (1900 probes) (Figure 1). Samples clustered into two main branches with vehicle and APAP treated mice on one branch, and IFN-β and IFN-β+APAP treated mice on the other. There was some clustering within time points, but the analysis revealed that APAP causes very little change in overall gene expression, while IFN-β and IFN-β+APAP treatments induce similar changes in transcript levels.

The 1900 probes that were considered significantly different from vehicle are displayed in Table S1. Of these, 784 probes which have a minimum difference of at least 1.5 fold from vehicle are summarized according to specific treatment and time groups (Table 1). This analysis revealed that very few genes are significantly affected by APAP alone at either 1.5 h (8 genes) or 4 h (2 genes). IFN-β demonstrated a much greater effect at both early and late time points with 165 and 133 probes detecting altered transcript levels respectively. Relative to the IFN-β treatment group, the combined treatment (IFN-β + APAP) resulted in a large increase (3 fold) of the number of genes with FDR p-values<0.05 at both 1.5 h and 4 h time points, suggesting that APAP significantly alters the gene expression pattern induced by interferon treatment (Table 1). At 1.5 hours the transcriptional response is balanced or up-regulated in IFN-β and IFN-β+APAP groups while at four hours the majority of transcripts were down-regulated in both sets. Previous studies have demonstrated similar temporal effects on transcription induced by APAP or IFN-β alone [33], [35]. The gene lists created following microarray analysis largely agreed with previously published results following recombinant interferon-β treatment [35], [36]. The same was not true for APAP alone. Where we found very few genes responsive to APAP others have demonstrated large number of gene expressed following similar treatments [33]. Likely factors contributing to this include the age of the CD-1 mice employed herein as well as the conservative hierarchical approach employed which would not have counted genes which showed a large degree of variability between sample sets.

**Table 1:** Probes that detected significantly (FDR ≤0.05) changed transcript levels and had a 1.5-fold difference from the vehicle control.

	Total		Up-regulated		Down-regulated	
	1.5 h	4 h	1.5 h	4 h	1.5 h	4 h
**APAP**	8	2	2	0	6	2
**IFN-β**	165	133	79	48	86	85
**IFN-β + APAP**	467	458	314	103	153	355

The number of probes that detect a significant change in the transcripts relative to the vehicle control is displayed. Table 1 displays total probes.

To examine the specific effects of APAP on IFN-β induced gene expression we collated all IFN-altered genes (Table S1) with a 1.5 fold or greater change and compared the response between IFN-β and IFN-β+APAP treatment sets. At 1.5 hours post-treatment 165 genes have significantly altered expression, 93 (52%) of which, have lower expression (less-positive or more negative) in the IFN-β+APAP treatment group. Specifically, 51 out of the 79 (65%) up-regulated genes and 42 of the 86 (49%) down-regulated genes have lower expression in the combined treatment. This pattern is accentuated at the 4 h time point where of the 133 genes altered by IFN-β, 110 (83%) have lower-expression, including 45 of 48 (94%) up-regulated genes and 65 of 85 (76%) down-regulated genes, in IFN-β+APAP co-treated mice.

To look at those genes with the largest differences we curated the list of significantly expressed IFN-β genes (Table S1) for those that showed at least a 1.5 fold-difference between IFN-β and IFN-β+APAP treatment sets (Table 2). This analysis highlighted several treatment effects. At 1.5 hours 16 of 79 (20%), while at 4 hours, 12 of 48 (25%), of IFN-β up-regulated genes, were differentially expressed by at least 1.5-fold in IFN-β+APAP treated mice. In all cases expression was lower in the co-treated mice. Five genes were common between these two time points, Ifit-3, Isg15, Oasl1, Zbp1 and a predicted gene EG634650 (Table 2). Amongst the 85 and 86 genes down-regulated by IFN-β at the 1.5 and 4 h time sets respectively only four genes at each time point demonstrated a 1.5-fold difference when mice received both IFN-β+APAP (Table 2). The four genes at the 1.5 hour time point were elevated (less down-regulated) while the four genes observed at 4 hours were more down-regulated in the IFN-β+APAP treatment group and no genes were in common between the two time points. This data suggests while there may be a specific effect of APAP on several genes up-regulated by IFN-β, the predominant effect is a broad, small depression of transcription. As we observe in Table1, this broad effect coincides with an overall global dampening of transcription in IFN-β+APAP treated samples.

**Table 2:** List of genes altered by IFN-β with a 1.5 fold difference in co-treatment.

Lower Expression in co-treatment						
Time Set	Gene	Description	IFN-β 1.5 h	IFN-β + APAP 1.5 h	IFN-β 4 h	IFN-β + APAP 4 h
Both	Ifit3	Interferon-induced protein with tetratricopeptide repeats 3, [NM_010501]	**12.79**	**5.8**	**49.55**	**22.85**
Both	Isg15	ISG15 ubiquitin-like modifier,[NM_015783]	**9.73**	**5.71**	**20**	**12.3**
Both	Oasl1	2′–5′ oligoadenylate synthetase-like 1, [NM_145209]	**5.19**	**3.11**	**13.29**	**7.33**
Both	Zbp1	Z-DNA binding protein 1, [NM_021394]	**3.72**	2.25	**13.1**	**6.7**
1.5	Rsad2	Radical S-adenosyl methionine domain containing 2, [NM_021384]	**5.61**	3.56	**12.85**	**10.02**
4	Usp18	Ubiquitin specific peptidase 18, [NM_011909]	**4.28**	3.12	**12**	**7.85**
1.5	Cxcl10	Chemokine (C-X-C motif) ligand 10, [NM_021274]	**14.21**	**7.52**	**9.75**	**8.85**
1.5	Ifi47	Interferon gamma inducible protein 47, [NM_008330]	**3.87**	2.46	**6.88**	**4.87**
Both	EG634650	Predicted gene, EG634650, [NM_001039647]	**4.78**	2.29	**6.52**	**4.09**
1.5	Socs1	Suppressor of cytokine signaling 1, [NM_009896]	**6.22**	**4.11**	**5.43**	**5.2**
1.5	Parp14	Poly (ADP-ribose) polymerase family, member 14, [NM_001039530]	**3.83**	**2.45**	**4.35**	**3.99**
4	2310016F22Rik	RIKEN cDNA 2310016F22 gene, [NM_173743]	1.03	−1.5	**3.94**	**1.73**
1.5	Mx1	Myxovirus (influenza virus) resistance 1, [NM_010846]	**3.6**	2.08	**3.42**	**3.12**
4	Nmi	N-myc (and STAT) interactor, [NM_019401]	1.17	−1.1	**3.09**	**1.79**
4	Serpina6	Serine (or cysteine) peptidase inhibitor, clade A, member 6, [NM_007618]	−1.05	1.12	**2.9**	1.53
4	Blnk	B-cell linker, [NM_008528]	−1.01	−1.34	**2.58**	1.19
4	Batf2	Basic leucine zipper transcription factor, ATF-like 2, [NM_028967]	2.09	1.54	**2.16**	1.43
1.5	Adm	Adrenomedullin, [NM_009627]	**2.02**	1.35	**2.11**	**1.81**
4	Myo5b	Myosin Vb, [NM_201600]	−1.22	−1.26	**2**	−1.02
1.5	Zbtb5	Zinc finger and BTB domain containing 5, [NM_173399]	**1.77**	1.11	1.28	1.1
1.5	Olfr578	Olfactory receptor 578, [NM_147115]	**2.29**	1.15	1.11	1.1
1.5	D330038O06Rik	RIKEN cDNA D330038O06 gene, [NM_177899]	**6.06**	1.47	−1.1	−1.01
1.5	Rnf186	Ring finger protein 186, [NM_025786]	**2.09**	1.37	−1.4	−1.39
4	Mtmr4	Myotubularin related protein 4, [NM_133215]	−1.02	1.03	**−1.63**	**−2.76**
4	Mettl7b	Methyltransferase like 7B, [NM_027853]	−1.13	−1.2	**−1.69**	**−2.66**
4	Grb7	Growth factor receptor bound protein 7, [NM_010346]	**−1.81**	**−1.95**	**−1.94**	**−3.12**
4	Fasn	Fatty acid synthase, [NM_007988]	**−3.19**	**−3.58**	**−2.42**	**−3.72**

Elevated Expression in co-treatment						
Time Set	Gene	Description	IFN-β 1.5 h	IFN-β + APAP 1.5 h	IFN-β 4 h	IFN-β + APAP 4 h
1.5	Hsd3b3	Hydroxy-delta-5-steroid dehydrogenase, 3 beta- and steroid delta-isomerase 3, [NM_001012306]	**−2.65**	−1.44	−1.23	−1.54
1.5	Dbp	D site albumin promoter binding protein, [NM_016974]	**−2.47**	−1.48	**−2.27**	**−2.13**
1.5	G6pc	Glucose-6-phosphatase, catalytic, [NM_008061]	**−10.51**	**−5.37**	−2.25	−1.75
1.5	EG624219	Predicted gene, EG624219, [NM_001080940]	**−2.31**	−1.44	1.56	−1.43

**Time Set** indicates the time point at which a 1.5-fold or greater change is observed between IFN and IFN+APAP samples. Values are the relative expression level of treatment compared to vehicle control. **Bolded** numbers indicate FDR <0.05.

Semi-quantitative real time RT-PCR was performed on the liver RNA samples harvested from the treated mice in order to confirm the expression levels of several genes identified using the Agilent 22 k microarrays (Table 3). These results confirmed the pattern of expression observed including the significant changes in the expression observed between treatment groups.

**Table 3:** Confirmation of selected genes using RT-PCR.

Encoded Protein/Gene	Symbol	Microarray			RT-PCR		
		APAP	IFN-β	IFN-β+APAP	APAP	IFN-β	IFN-β+APAP
Heme oxygenase (decycling) 1	Hmox1	2.7	−1.5	2.3	9.6	−3.8	12.1
Keratin 18	Krt18	−1.3	1.1	−2.0	−1.7	1.1	−2.0
B-cell linker	Blnk	−1.0	2.6	1.2	−2.3	10.1	6.1
2′–5′ Oligoadenylate synthetase 2	Oas2	1.0	1.8	1.3	−1.3	26.8	13.0
Interleukin-1 receptor-associated kinase 2	Irak2	−1.1	−1.0	−1.6	−1.5	−2.8	−4.0
EG634650	EG	−1.2	6.5	4.1	−3.6	95.3	24.7
Interferon-induced protein with tetratricopeptide repeats 3	Ifit3	−1.1	49.6	29.9	−3.1	376.9	109.2
Interferon Stimulated Gene 15 ubiquitin-like modifier	Isg15	−1.3	20.0	12.3	−1.2	245.1	96.1
2′–5′ Oligoadenylate synthetase-like 1	Oasl1	−1.2	13.3	7.3	−1.8	298.0	80.6
Z-DNA binding protein 1	Zbp1	−1.2	13.1	6.7	−2.6	113.5	71.3

The absolute fold-change was determined using semi-quantitative RT-PCR on 5 genes from the 4 hour time point and compared to the microarray analysis. In all cases numbers are relative to vehicle control.

### Ontology Analysis

In order to explore common themes of affected genes we employed DAVID (**D**atabase for **A**nnotation, **V**isualization and **I**ntegrated **D**iscovery, NIAID), an on-line resource that identifies enrichment of genes with specific gene ontology (GO) terms [27], [28]. Table 4 displays the results of this analysis, and includes biological pathways from the top annotation clusters enriched in each treatment group by analysis of the probes with fold-changes greater than 1.5. At 1.5 hours, genes down-regulated by APAP treatment were enriched in one functional group representing lipid biosynthesis. Genes up-regulated by IFN-β treatment at this time point clustered to several functional groups all involved in immune responses, while down-regulated genes grouped strongly with cellular biosynthesis and metabolic processes. Genes up-regulated by IFN-β+APAP co-treatment were over-represented in 8 functional groups predominately involved in cell signalling and development, while down-regulated genes clustered strongly with cellular metabolism and biosynthesis pathways (Table 4). No clusters of terms related to immune responses (such as immune response, response to virus, defence response, etc.), were enriched amongst the up-regulated genes that exhibited at least a 1.5 fold change in expression. However, when the IFN-β+APAP set was repeated using only probes showing≥2 fold-changes relative to vehicle we detected enrichment in one functional group, a GO cluster of immune response pathways (data not shown). Therefore, many of the observed differences between IFN-β and the combined treatment group are likely due to additional genes up-regulated in the IFN+APAP set as opposed to the specific absence of immune related genes.

**Table 4:** Summary of Gene Ontology Analysis.

Treatment Group	Reg.	Annotation Clusters	Top Biological Processes	p-VALUE	Matched Genes	Gene List
APAP 1.5 hrs	−1.5	1	GO:0008610∼lipid biosynthetic process	0.0028	3	HSD3B3, HSD3B5, DGAT2
IFN-β 1.5 hrs	+1.5	4	GO:0006955∼immune response	2.63E-09	14	IFI204, IFIT1, IRF1, SERPINA3G, IFIT3, MX1, CCL2, GADD45G, MX2, ISG15, IL27RA, CXCL10, OASL1, GBP5
			GO:0009615∼response to virus	0.000948	4	MX1, MX2, RSAD2, ISG15
			GO:0006915∼apoptosis	8.30E-02	6	GADD45G, SERPINA3q, PHLDA1, CASP4, DAXX, IFI204
	−1.5	10	GO:0008610∼lipid biosynthetic process	3.40E-04	8	AACS, ACLY, DGAT2, ELOV6, FASN, HSD3B3, HSD3B5, HSD3B6
			GO:0008202∼steroid metabolic process	1.70E-04	7	CLN8, CYP7A1, HSD3B3, HSD3B5, HSD3B6, NR1H4, SREBF1
			GO:0042445∼hormone metabolic process	7.80E-03	4	ALDHA1, HSD3B3, HSD3B5, HSD3B6
IFN-β+APAP 1.5 hrs	+1.5	8	GO:0007186∼G-protein coupled receptor protein signaling pathway	1.74E-07	47	htr5a, bdkrb1, celsr2, gpr44, gprc5d, galr3, LPHN3, MRGPRB5, MRGPRG, MC2R, MC4R, OLFR544, OLFR100, OLFR104, OLFR1198, OLFR1226, OLFR1248, OLFR1280, OLFR1307, OLFR1395, OLFR148, OLFR166, OLFR195, OLFR225, OLFR304, OLFR457, OLFR508, OLFR524, OLFR549, OLFR570, OLFR59, OLFR656, OLFR711, OLFR74, OLFR894, OLFR971, OLFR996, OLFR128, ACR, A2RY5, RPH3AL, OLFR112, SSTR4, SSTR5, TAC2, UTS2R, V1RA7, V1RE12
			GO:0006508∼proteolysis	0.0039818	19	ATG4D, CASP4, CTSG, CTRB1, CCDC9, GZMK, ISG15, MMP17, PGM5, ACR, PCSK2, KLK8, PRSS22, PRSS3, CTRL, ZFP3, ZMPSTE24, RIKEN CDNA 2600011L02
			GO:0048730∼epidermis morphogenesis	3.10E-02	4	SPRR2B, SPRR2Q, RUNX3, GPRC5D
	−1.5	14	GO:0044238∼primary metabolic process	1.40E-04	56	APOA5, ANGPTL4, CAR3, CAR5A, CSNK1G3, BCO16495, ZFP768, CLN8, C1QA, CCND1, DGAT2, DAK, ELOVL6, EIF2B4, EIF3I, FOXA3, INHBA, MAP2K6 NDUFA3, SREBF1, SNRK, RPS15, PER2
			GO:0009991∼response to extracellular stimulus	4.10E-04	4	ANGPTL4, FOXA3, PPAN, SREBF1,
			GO:0044249∼cellular biosynthetic process	4.81E-05	20	ALAS1, AARS, FASN, NDUFA13, GSS, RPL7L1,GAMT, PDXK CAR3, CAR5A, GPI1, MAT2A, DGAT2, SDS, HMG20B, HSD3B5, GCK, RPS15, ELOVL6, EIF2B4
APAP 4 hrs		0	0	N/A	N/A	N/A
IFN-β 4 hrs	+1.5	4	GO:0006955∼immune response	8.68E-08	11	OASL1, CXCL10, DBNL, GBP5, IFI204, ISG15, IFIT1, IFIT3, MX1, MX2, SERPINA3G
			GO:0009615∼response to virus	4.13E-04	4	MX1, MX2, RSAD2, ISG15
	−1.5	3	GO:0006629∼lipid metabolic process	9.40E-05	12	ALDH8A1, AMACR, ACLY, DGAT2, ELOVL3, FASN, INSIG2, MGLL, MTMR4, NR1H4, 4632417N0RRIK, SEC14L2
IFN-β+APAP 4 hrs	+1.5	3	GO:0006955∼immune response	2.30E-06	13	OASL1, CXCL10, GBP5, IFI204, IRF1, IL27RA, ISG15, IFIT1, IFIT3, MX1, MX2, SERPINA3G, UNC93B1
			GO:0009615∼response to virus	0.0019098	4	MX1, MX2, RSAD2, ISG15
	−1.5	12	GO:0010467∼Gene Expression	1.20E-04	75	MRPL49, FOXA3, MRPL36, LSR, SMARCB1, RNF20, SREBF1, HES6, SIRT7, RPL19, CNBP, PYGO2, ING4, POLR2I, NFIC, FTSJ3, CPSF1, NCOA5, MRPL16, 2610209M04RIK, POLR2C, SMARCD2, PSEN2, POLR2J, SART1, PRPF31, ZBTB7A, ZFPM1, CSTF1, ZFP768, EIF5B, NR1H4, NCOR2, POP5, SEC14L2, SUPT6H, HDGFRP2, LARS, RPL7L1, 5730449L18RIK, USP39, TUFM, CHERP, EIF4A3, PHF1, POLRMT, DBP, SF4, TRAK1, MRPL43, SUV420H2, EDF1, PER2, LSM7, GTF2E1, ZFP655, SARS, CPSF3, TSEN54, CHD3, MED6, EIF1B, DEDD2, MEF2D, MLH1, GTF2E2, CREB3L3, MRPS16, TFAM, IRF3, SSRP1, CIAO1, SNAPC4, SART3, ID2, EIF2B4,
			GO:0008610∼lipid biosynthetic process	0.0012961	12	AACS, ACLY, CNBP, DGAT2, D5WSU178E, ELOVL3, ELVOL6, FASN, HSD3B5, MLYCD, PCYT2, 4632417N05RIK, SEC14L2
			GO:0006397∼mRNA processing	0.0051249	12	CPSF1, CPSF3, CSTF1, EIFA3, LSM7, MLH1, PRPF31, 2610209M04RIK, 5730449L18RIK, SF4, SART1, USP39

**Treatment Group**: Treatment group analyzed; **Reg.**: direction and minimum magnitude of analyzed genes, **Annotation Clusters**: Total number of functional annotation clusters generated based on the grouping of gene ontology terms identified according to biological processes, cellular components and molecular functions. **Top Biological Processes**: Top GO biological processes identified; ***p*** **-VALUE**: The statistical significance of this grouping relative to random chance as measured by the EASE test. The lower the score the more unlikely this clustering is due to chance; **Matched Genes**: The number of genes contributing to this GO term; **Gene List**: List of genes that contribute to the GO biological process.

At the 4 h time point genes up-regulated after IFN-β treatment clustered with two GO biological processes, both involved in the host immune response. Only one biological process group, lipid metabolism, was enriched by genes down-regulated during IFN-β treatment. GO pathways represented by genes up-regulated by IFN-β+APAP treatment were identical to the IFN-treatment group. Genes down-regulated by IFN-β+APAP clustered into 27 functional groups which were overwhelmingly associated with cellular metabolism and gene expression pathways, such as RNA processing or translation (Table 4). Overall these data suggest that the largest effect of APAP during IFN-β treatment is to cause a short-term up regulation of sensory and developmental genes but by 4 hours repress genes involved in transcription and biosynthesis.

### Gene Set Enrichment Analysis

Evaluation of microarray data using MAANOVA statistics with an FDR-adjusted *p*-value of 0.05 is a conservative approach for the identification of differential gene expression. It has been suggested that such an approach can result in a large number of false negative findings and the loss of valuable data [37]. Furthermore, DAVID doesn't take into consideration gene expression levels limiting analysis possible given the broad similarity of effect (Figure 1). Therefore, we re-evaluated some of our results using the Gene Set Enrichment Analysis (GSEA) software package to improve our understanding of the gene sets or pathways that responded to treatment. As the overall results between the two time points were similar we chose to limit this analysis to the 4 hour dataset. The results of the GSEA analyses are shown in Table 5 at an FDR<0.1 with the full results shown in Table S2. APAP treatment alone showed no significant differences from that of the vehicle control group, in broad agreement with our previous analyses. GSEA analysis of the IFN-β treatment group identified 23 pathways that were up-regulated relative to vehicle control (Table 5). Of these 23 pathways, 15 are directly related to the immune response, 3 to proliferation and the remainder to signal transduction, protein transport and protein modification processes. IFN-β treatment also led to the down regulation of 4 pathways involved in transcription/translation and DNA repair. GSEA analysis of IFN-β+APAP versus vehicle (I.A. v V.) revealed a significant deviation from the IFN-β induced response. Genes up-regulated by IFN-β+APAP versus vehicle were involved in 2 biological pathways both related to the immune response related (Table 5). No pathways were identified as down-regulated in the I.A. datasets.

**Table 5:** Gene Set Enrichment Analysis.

Comp.	Reg.	Gene set name	Genes	NES	FDR
A. vs V.	+				
A. vs V.	−				
I. vs. V.	+	DEFENSE_RESPONSE	46/211	1.98	0.021
	+	I_KAPPAB_KINASE_NF_KAPPAB_CASCADE	27/100	1.92	0.032
	+	HUMORAL_IMMUNE_RESPONSE	8/28	1.91	0.028
	+	RESPONSE_TO_OTHER_ORGANISM	17/61	1.89	0.03
	+	REGULATION_OF_I_KAPPAB_KINASE_NF_KAPPAB_CASCADE	23/82	1.88	0.026
	+	IMMUNE_RESPONSE	40/198	1.88	0.025
	+	INFLAMMATORY_RESPONSE	20/116	1.83	0.043
	+	MEIOSIS_I	3/17	1.83	0.038
	+	MULTI_ORGANISM_PROCESS	26/114	1.82	0.036
	+	POSITIVE_REGULATION_OF_I_KAPPAB_KINASE_NF_KAPPAB_CASCADE	21/78	1.82	0.033
	+	MEIOTIC_RECOMBINATION	3/15	1.79	0.043
	+	MEIOTIC_CELL_CYCLE	8/31	1.76	0.059
	+	POSITIVE_REGULATION_OF_SIGNAL_TRANSDUCTION	22/113	1.75	0.065
	+	JAK_STAT_CASCADE	10/28	1.75	0.065
	+	RESPONSE_TO_VIRUS	13/40	1.74	0.068
	+	CYTOKINE_AND_CHEMOKINE_MEDIATED_SIGNALING_PATHWAY	5/16	1.73	0.069
	+	PEPTIDYL_TYROSINE_MODIFICATION	3/27	1.71	0.077
	+	RESPONSE_TO_BIOTIC_STIMULUS	22/91	1.7	0.082
	+	DEFENSE_RESPONSE_TO_BACTERIUM	5/17	1.7	0.078
	+	IMMUNE_SYSTEM_PROCESS	42/281	1.69	0.088
	+	RESPONSE_TO_WOUNDING	24/169	1.68	0.086
	+	REGULATION_OF_T_CELL_ACTIVATION	6/25	1.67	0.096
	+	TRNA_METABOLIC_PROCESS	7/19	1.66	0.099
I. vs. V.	−	TRANSLATIONAL_INITIATION	10/25	−1.8	0.051
	−	REGULATION_OF_TRANSLATIONAL_INITIATION	8/20	−1.84	0.055
	−	NUCLEOTIDE_EXCISION_REPAIR	8/18	−1.81	0.057
	−	TRANSCRIPTION_FROM_RNA_POLYMERASE_III_PROMOTER	9/16	−1.85	0.09
A.I. vs V.	+	DEFENSE_RESPONSE	52/211	1.96	0.062
	+	RESPONSE_TO_OTHER_ORGANISM	12/61	1.92	0.073
A.I. vs V.	−				
A.I. vs. I.	+				
A.I. vs. I.	−	GOLGI_VESICLE_TRANSPORT	27/45	−2.43	0.000
	−	ER_TO_GOLGI_VESICLE_MEDIATED_TRANSPORT	10/16	−2.24	0.000
	−	SECRETORY_PATHWAY	25/78	−2.18	0.000
	−	MRNA_PROCESSING_GO_0006397	28/65	−2.10	0.002
	−	SECRETION_BY_CELL	28/105	−2.07	0.002
	−	RNA_SPLICING	30/81	−1.98	0.010
	−	MRNA_METABOLIC_PROCESS	29/73	−1.97	0.010
	−	RNA_PROCESSING	47/153	−1.96	0.010
	−	RNA_SPLICING__VIA_TRANSESTERIFICATION_REACTIONS	15/31	−1.95	0.010
	−	I_KAPPAB_KINASE_NF_KAPPAB_CASCADE	34/100	−1.85	0.033
	−	SPLICEOSOME_ASSEMBLY	9/18	−1.85	0.034
	−	INTRACELLULAR_TRANSPORT	82/255	−1.82	0.041
	−	TRANSCRIPTION_INITIATION_FROM_RNA_POLYMERASE_II_PROMOTER	6/18	−1.80	0.048
	−	TRANSCRIPTION_INITIATION	7/24	−1.74	0.082

A.  = APAP, I.  = IFN-β, V.  = Vehicle, I.A.  = IFN-β+APAP. **Comp**.  = Comparison, **Reg.**  = Regulation and ‘+’ indicates up-regulated and ‘−’indicates down-regulated., **Genes** = Genes Enriched/Total Genes in Set, **NES** = Normalized Enrichment Score, **FDR** = False Discovery Rate.

We also directly compared the IFN-β+APAP treatment group to the IFN-β treatment group (I.A.vs I.). We detected no positive enrichment of pathways in this comparison but did find 14 pathways that were down-regulated by IFN-β+APAP relative to IFN-β alone. These were predominately transcription and transportation related pathways in addition to the IκB/NF-κB cascade.

**Figure 2:**
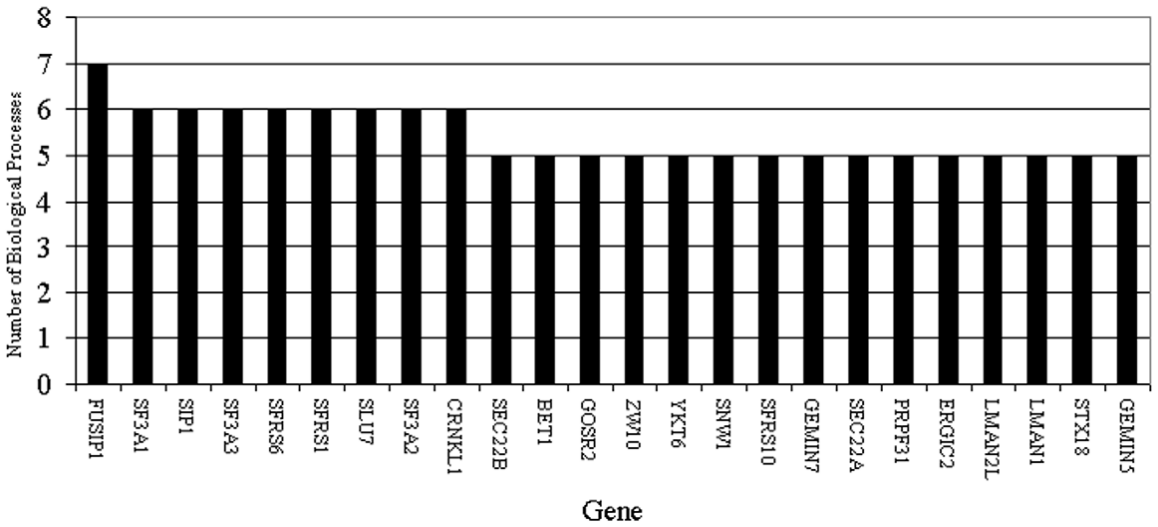
Leading edge analysis of genes downregulated by IFN-β + APAP treatment relative to IFN-β treatment at 4 h. A total of 178 genes contribute to the enrichment of 14 biological pathways down-regulated in the IFN-β + APAP treatment group relative to IFN-β alone. The 24 genes that participate in 5 or more of these pathways are shown.

Leading edge analysis was conducted on the gene sets enriched at FDR<0.1. The results for the analysis of IFN-β+APAP versus IFN-β are displayed in Figure 2. Leading edge analysis assumes that genes that contribute to multiple pathways will be of greater interest than those that only contribute to one pathway.

There are 178 genes that contribute to the enrichment of 14 down-regulated pathways when IFN-β+APAP is compared to IFN-β (Figure 2). Of these genes, 24 contribute to 5 or more pathways including Lman1, GosR1, Bet1, Ykt6, Sec22A, Ergic2, Zw10, Lman2L, Stx18, all of which are involved in cellular membrane trafficking events. Additional genes that were enriched in multiple biological pathways include Fusip1, Sip1 (Gemin2), Gemin5, Gemin7, Sf3A1, Sf3A2, Sf3A3, SfrS1, SfrS6, SfrS10, Slu7, Snw1, Prpf31 and Crnkl1 all of which are involved in RNA processing.

## Discussion

This study has demonstrated the effect of APAP on the murine hepatic response to IFN-β *in vivo* using a whole genome microarray. Over 1900 genes from the large liver lobe of mice were significantly responsive to treatment with IFN-β, APAP or IFN-β+APAP; of these 784 were differentially expressed at a level of 1.5 fold over vehicle control. The response to IFN-β in live animals supported previous observations made in cell culture [36]. Many genes were differentially expressed when APAP was co-administered with IFN-β. This was in contrast to the very few genes that were responsive to APAP alone. Cluster analysis revealed that APAP and control liver samples shared very similar expression profiles, while IFN-β and IFN-β+APAP profiles were highly correlated. However, the synergistic effect of APAP on the IFN-β transcriptional response was significant in the context of the magnitude of the IFN- β transcriptional response. It is likely that the synergistic effect we observe is due to the specific effect of APAP on gene expression rather than liver injury. No hepatotoxicity was detected and none was expected as 6–8 week old CD-1 mice have been shown to maintain liver activity in the presence of higher APAP doses than administered in this study [31], [32].

Of the genes significantly up-regulated by IFN-β treatment the majority were lower in IFN-β+APAP samples including 45 of the 48 genes significantly elevated 4 hours post-treatment. Significantly, 25% of these genes are diminished by 1.5 fold or greater. While a large number of genes down-regulated by IFN-β treatment are further diminished by APAP at this time point only 4 (5%) were reduced by 1.5-fold. There may be multiple factors governing the depression of the IFN-β response. Some genes up-regulated by IFN-β have significantly diminished expression following APAP administration suggesting a specific effect on one or several genes involved in IFN-β signalling. However, the observed effects are small on the majority of IFN-β-responsive genes and likely due to the negative effect that IFN-β+APAP has on genes involved in RNA processing and transcription.

Amongst the 27 IFN-β induced genes whose expression was diminished 1.5 fold or more by APAP co-treatment five genes, Ifit-3, Isg-15, Oasl1, Zbp1 and EG634650 or GBP11 are detected at both time points. Ifit-3 (Rig-G) is an RNA helicase induced via multiple signals including STAT2/IRF-9 and double-stranded RNA [38]. Ifit-3 is involved in the activation of cytosolic IRF-3 and NF-κB [39] as well as the down-regulation of c-Myc [40]. The down-regulation of c-Myc may be an important function of Ifit-3 as previous studies have demonstrated that c-Myc expression can impair the IFN response and in particular, suppress the NF-κB cascade [41], [42]. APAP has been demonstrated to induce c-Myc expression suggesting one means by which APAP may globally interfere with effects of recombinant IFN-β. Furthermore, APAP inhibits degradation of I-κB thus interfering with NF-κB activation [43] suggesting another means by which APAP could interfere with IFN signalling. A second inhibitor of c-Myc activity, Nmi, was significantly diminished at 4 h, although not at 1.5 h post-treatment (Table 2).

Isg15, an ubiquitin–like enzyme had substantially diminished expression following co-treatment at both time points, while the Isg-15 specific protease, Usp18 was only diminished by more than 1.5-fold at 4 hours. Isg15 and Usp18 are interferon-induced ubiquitin conjugating/deconjugating proteins which down-regulate the JAK/STAT pathway in a negative feedback loop [44]. In addition to targeting IFN-induced proteins, such as Ifit-3, Mx1 and PKR, Isg15 also targets proteins from a range of other pathways for degradation including those involved in RNA splicing, transcription and translation [45].

Oasl1, is a member of the 2′–5′ oligoadenylate synthetase family, however it lacks obvious OAS activity. Oasl1, however, does possess antiviral properties against some RNA viruses, although the specific mechanism by which it operates is unclear [46].

Zbp1 is a cytosolic “danger” sensor that activates IRF3 and NF-κB pathways in response to cytoplasmic DNA [47]. Zbp1 activation of IRF3 is dependent on the recruitment of TBK-1 (TANK-binding kinase-1) and an IκB kinase, both of which are involved in NF-κB activation.

The predicted sequence EG634650 has been identified as belonging to the guanylate binding protein family, one of three types of GTPases induced by IFN sub-types. Identified family members have antiviral effects in certain cell lines and can be induced by both IRF-1 and STAT1 [48].

The depression of genes that can positively or negatively regulate IFN-signalling in addition to downstream IFN targeted genes suggest that lower overall signal transduction occurs when IFN-β is administered with APAP. No differences, however, were observed in the expression of IFNAR1 or 2, STAT1, STAT2 or STAT3. One explanation may be that while the expression of these genes remains constant the activity of one or more of the STAT proteins is depressed in the presence of APAP.

Ontology analysis highlighted differences in biological processes that were affected by the individual treatments relative to the combined treatment. An important example is the response of G-protein coupled receptors (GPCRs). The most highly represented responsive genes in the DAVID analysis were GPCRs and these were exclusively enriched in the combined treatment group at 1.5 hours. The majority of these GPCRs are components of the odorant sensory system. Olfactory receptors (ORs) are the largest mammalian gene family with over 1000 representatives in the murine genome [49]. ORs are expressed in a variety of cells and tissues but their contributions to signalling outside of the odorant system are largely undefined [50], [51]. Several studies have suggested that ORs may be involved in chemosensory functions [51], [52]. The induction of OR mRNAs has been observed in previous studies examining the transcriptional response of C57BL/6 mice to APAP-induced liver injury [53]. An early up-regulation of chemosensory receptors following IFN-β+APAP treatment may reflect an initial response to cellular stress or conflicting stimuli.

By 4 hours post-treatment GPCRs were no longer identified among GO terms. All of the OR GPCRs detected at the earlier time point were considered up-regulated (FDR<0.05) at 4 hours, but the majority remained below the 1.5-fold selection threshold. This may coincide with an overall decrease in gene expression following APAP treatment. By four hours post-treatment the list of GO biological processes clustered through DAVID analysis were predominately involved in mRNA transcription, processing and translation. The down-regulation of genes involved in transcription and mRNA processing could contribute to the observed dampened transcriptional response.

A similar picture is provided by the GSEA analysis. Consistent with the GO terms clustered using DAVID IFN-β treatment predominately up-regulates genes in immune response pathways. IFN-β treatment down-regulates gene sets involved in transcription and translation. It is notable that few pathways are identified at an FDR of 0.1 in the combined treatment group compared to vehicle control, given how many significant differences we observed in the gene-to-gene analysis. This is partly explained when we extend our analyses to a higher FDR level of 0.25. In this case we observe the positive enrichment of 6 additional immune related pathways and extensive negative enrichment of pathways related to protein synthesis, RNA transcription, RNA processing, protein transport and protein modification in the IFN-β+APAP group (data not shown).

When we directly compared the IFN-β+APAP treatment group to IFN-β we found no pathways were up-regulated in the combined treatment relative to IFN-β alone. We did observe the down-regulation of 13 gene sets related to RNA transcription/processing or protein transport. Additionally, the IκB/NF-κB pathway was down-regulated due to the negative enrichment of 34 genes, four of which (MyD88, Trif, Traf2 and IκBKε) are also involved in the activation of interferon regulatory factors.

Leading edge analysis (LEA) did not find any genes involved in the NF-κB pathway. This was not surprising as LEA searches the enriched pathways for genes that appear multiple times and 13 of the 14 pathways down-regulated by IFN-β+APAP are involved in either RNA processing or membrane trafficking events. The observed comprehensive decrease in genes involved in RNA processing suggests that overall protein synthesis is likely to be diminished in hepatic cells during co-treatment. Additional evidence for a decrease in protein synthesis may be provided by the observed down-regulation of genes involved in retrograde trafficking. One of the principle functions of the retrograde trafficking system is to ensure ER-homeostasis and to retrieve Golgi and pre-Golgi proteins. A reduction in protein synthesis would decrease the need for retrograde trafficking components.

APAP modifies IFN-β-induced gene expression patterns in the liver by activating genes involved in chemosensory or signal transduction pathways and down-regulating genes involved in cellular metabolism. The vast majority of genes that are differentially regulated by IFN-β treatment alone remain transcriptionally active during co-treatment. However, a marked shift was observed in the Gene Ontology (GO) terms clustered by DAVID. This is due in part to the application of a minimum 1.5-fold change but is also due to an increase in the total number of genes regulated by IFN-β+APAP as compared to IFN-β alone. DAVID calculates an enrichment score based on a comparison between the number of genes that cluster to a specific GO term relative to the number of genes that would be expected to cluster to that term based on random chance. For example, the GO category apoptosis has an enrichment score of 1.02 based on the up-regulation of 5 out of 70 genes by IFN-β treatment at 1.5 h. The same GO term, apoptosis, only has an enrichment score of 0.62 based on the up-regulation of 12 out of 263 genes in the IFN-β+APAP group. Therefore, despite the fact that the same genes appear in both treatment groups the larger number of non-apoptosis related genes that are also activated in the co-treatment group result in a low enrichment score for the GO term apoptosis.

Another factor not considered by DAVID when analyzing genetic data are changes in the fold-expression of IFN- β responsive genes upon co-treatment with APAP. For example, 121 genes are responsive in the co-treatment group and the IFN-β group at 4 hours. Of these, 110 have decreased expression levels, though their categorical pattern of regulation remains the same (less-positive or more-negative). This suggests that APAP modifies the IFN-β response in a global and systemic fashion not considered by DAVID. The mechanism of APAP-induced down-regulation is unclear; it may involve one or more transcription factors normally induced or repressed by IFN-β as well as the overall down-regulation of genes whose products function in transcription and translation.

This study examined gene expression profiles from the whole murine liver. Changes in less abundant cell populations may not have been detected and it is impossible to attribute the observations to any one cell type. Previous studies combining IFN-α and APAP found no evidence for a reduction in the antiviral or anti-proliferative response of immortalized cell lines or isolated PBMCs [19], [54]. The global transcriptional change in a heterogeneous cell population observed here may be driven by different cell types. Alternatively, the *in vitro* antiviral response may not be sensitive enough to detect the changes observed in this study [19], [54]. Our data indicate that APAP effects quantitative but not qualitative changes to the expression patterns induced by IFN-β. As such, APAP is more likely to lower the efficacy of IFN-β treatment as opposed to abrogate a desired endpoint. Overall, this data fits with a recent study which demonstrated that children who were treated prophylactically with acetaminophen during vaccination had diminished antibody titers, although the majority had successful vaccine responses [13]. The results presented here suggest that a dampened NF-κB response in response to APAP co-treatment may have had a role in this outcome.

In conclusion, we demonstrate that APAP administered during recombinant IFN-β treatment induces a broad change in the transcriptional profile of hepatic genes. These expression changes have the potential to modify the IFN-β response and may explain occasional APAP-mediated hepatotoxicity observed in patients when recombinant interferon is combined with a tertiary treatment [55]. More importantly, this research suggests that the casual administration of APAP alongside IFN-β may result in unintended dampening of the transcriptional response and may reduce the efficacy of IFN-β treatment.

## Supporting Information

Table S1 A summary of all the significantly altered probes detected at an FDR of <0.05.
(0.73 MB XLS)

Table S2 A.  = APAP, I.  = IFN-β, V.  = Vehicle, I.A.  = IFN-β+APAP. Comp.  = Comparison, Reg.  = Regulation and ‘+’ indicates up-regulated and ‘−’ indicates down-regulated, Genes = Genes Enriched/Total Genes in Set, NES = Normalized Enrichment Score, FDR = False Discovery Rate.
(0.04 MB XLS)
